# Pair-bonding leads to convergence in approach behavior to conspecific vocalizations in California mice (*Peromyscus californicus*)

**DOI:** 10.1371/journal.pone.0255295

**Published:** 2021-08-12

**Authors:** Nathaniel S. Rieger, Patrick K. Monari, Kamryn Hartfield, Juliette Schefelker, Catherine A. Marler

**Affiliations:** 1 Department of Psychology, University of Wisconsin-Madison, Madison, Wisconsin, United States of America; 2 Department of Psychology and Neuroscience, Boston College, Chestnut Hill, Massachusetts, United States of America; University of Missouri Columbia, UNITED STATES

## Abstract

Pair-bonding allows for division of labor across behavioral tasks such as protecting a territory, caring for pups or foraging for food. However, how these labor divisions are determined, whether they are simply intrinsic differences in the individual’s behavior or a coordinated behavioral response by the pair, remains unknown. We used the monogamous, biparental and territorial California mouse (*Peromyscus californicus*) to study how behavioral approach to an aggressive vocal stimulus in a novel environment was affected by pair-bonding. Using a three-chambered vocal playback paradigm, we first measured the amount of time individuals spent in close proximity to aggressive bark vocalizations. We found that animals could be categorized as either approachers or avoiders. We then paired individuals based on their initial approach behavior to an opposite sex individual who displayed either similar or different approach behaviors. These pairs were then retested for approach behavior as a dyad 10–11 days post-pairing. This test found that pairs showed convergence in their behavioral responses, such that pairs who were mismatched in their approach behaviors became more similar, and pairs that were matched remained so. Finally, we analyzed the ultrasonic vocalizations (USV) produced and found that pairs produced significantly more USVs than individuals. Importantly, increased USV production correlated with increasing behavioral convergence of pairs. Taken together, this study shows that pair-bonded animals alter their approach behaviors to coordinate their response with their partner and that vocal communication may play a role in coordinating these behavioral responses. Overall, our findings indicate that pair-bonding generates an emergent property in pairs, adjusting their combined approach behavior towards a new aggressive stimulus representing a potential challenge to the bonded pair. Such findings may be broadly important for social bonding in other social systems.

## Introduction

Within pair-bonding species, reproductive success is influenced by partners’ behavioral compatibility [1]. This compatibility can be due to either behavioral plasticity or assortative mating and, as such, studying how individual behavioral responses change after pair-bonding can give us important insights into how social context alters the behavior and coordination of animals [2–4]. It is vital for pairs to coordinate their behavior in a way that maximizes their likelihood to protect their territory and raise their offspring in the most efficient manner possible [5]. However, whether these roles are determined by intrinsic behavioral traits of the individual pair members, or is determined through coordination within the pair and alterations of individual behavior is not well understood [6–9]. As such, it is of great interest to know whether animals maintain their individual behavioral responses to stimuli after pairing or if being a part of a pair leads to changes in these behaviors to better coordinate responses, such as joint avoidance or approach to an aversive, aggressive stimulus. Such coordination in behavior may also be relevant for species with social bonds beyond that of male-female pairings and beyond that of territorial defense.

One major advantage to pair-bonding is the ability to divide labor within tasks to maximize efficiency and increase success in breeding and survival. Across taxa, group living animals divide labor in a number of ways [10–15]. In pair-bonding species this is often done in a sex specific manner, for example, with females remaining close to the nest and caring for offspring, while males patrol the edges of a territory to defend against threats and forage for food [16–18]. However, in some species these roles are more fluid, with both males and females capable of completing all tasks required of the pair including territorial defense [19–23]. How this is achieved though remains unknown and is likely, to some extent, species specific.

In order to better understand the role of pair-bonding in division of labor and approach behavior we studied the monogamous, biparental and territorial California mouse (*Peromyscus californicus*). California mice are an interesting species in which to understand coordination of pair behavior because both males and females are capable of doing all necessary tasks, including defending territories and caring for pups [19, 20, 24, 25]. Individuals move from their natal territory, with females dispersing farther from the natal territory than males. Males typically set up a territory first, with the female joining a male to jointly defend an exclusive territory [26]. DNA fingerprinting indicates that this is a strictly monogamous species and both sexes are highly aggressive and both contribute significantly to the raising of offspring; males are, in fact, essential for maximizing weight and survival of offspring [27]. How these tasks are completed, particularly investigating intruders and defending territories varies depending on the pair. Specifically, California mice show differing strategies across pairs to investigate intruders together [19], while other pairs will have only one member (either male or female) investigate while the other remains close to the nest [13, 16]. Importantly, it is unknown whether these roles are determined solely by intrinsic traits of the individuals [28] or if there is behavioral coordination, constituting an emergent property of the pair that occurs within the pair to determine roles. Moreover, California mice produce a suite of ultrasonic vocalizations that appear to be important in signaling behavioral intent and pair communication [29–33]. In particular, California mice produce sustained vocalizations (SV) that can signal aggression towards outsiders in its shorter form [25] and affiliation within a pair bond in its longer form [32, 34, 35]. As such we were interested in testing how California mouse approach behavior and SV production when exposed to a simulated intrusion via playbacks would be altered by the formation of a pair bond and the presence of their mate.

In this study we hypothesized that individual, nonbonded California mice would show variation in their approach behavior towards an aversive stimulus and that pair-bonding would alter these responses. Further, we hypothesized that SV production would be consistent with the need to coordinate behaviors between pair members. To test this, we conducted a playback study in which individual mice were exposed to aggressive conspecific ‘bark’ calls [25, 30], short, high-amplitude calls that begin and end in the broadband range, that are used in defensive aggression; we then measured how much time the focal mouse spent investigating the call as defined by being in the chamber closest to the playback in a three chambered cage. Following individual testing we paired individuals in a 2 (high vs low approach) x 2 (male versus female) factorial design with an opposite sex partner that either shared (matched) or differed (mismatched) in their approach pattern and retested the pair after pair bonding in order to glean further insight into how pair-bonding affects individual and group behavior.

## Methods

### Animals

31 male and 31 female sexually naïve California mice (age 3–6 months, housed with 1–2 same-sex conspecifics) were tested for responses to unfamiliar conspecific bark playbacks. Mice were then selectively paired with an opposite sex partner 3–7 days following the initial (i.e., pre-pairing) playback test and housed together in a standard cage (48 x 27 x 16 cm). All animal housing cages were lined with aspen bedding, contained a nestlet and had Purina 5015^™^ mouse chow and water available *ad libitum*. All tests occurred between 1–3 hrs after the onset of the dark cycle in dim red light in housing maintained at 20–23° C on a 14:10 h light: dark cycle (lights on at 16:00 central standard time).

### Ethical statement

All animals were maintained according to the National Institute of Health *Guide for the care and use of laboratory animals*. All procedures were approved by the University of Wisconsin–Madison College of Letters and Sciences Institutional Animal Care and Use Committee (Protocol L005447). No animals were injured by any of the behavioral manipulations or assays.

### Apparatus

Testing occurred in Plexiglas cages (90 x 30 x 30 cm) lined with aspen bedding equally divided into three chambers (each 30 x 30 x 30 cm) with centrally located openings (11.5 x 11.5 cm) between chambers to allow for free movement. A speaker (Vifa Dynamic Ultrasound, 1–120 kHz range, Avisoft Bioacoustics, Berlin, Germany) was placed at each end of the three-chambered apparatus 45 cm from the center. Speakers were positioned outside of the apparatus against a closed mesh gate.

### Playback tracks

In a separate cohort of mice, barks were recorded from males and females housed under the same conditions as experimental mice to produce eight unique tracks (see below), that were assigned randomly to individuals. To avoid habituation and maintain consistency, no individual heard the same track more than once [35]. To induce calling to create playback tracks, individual male and female mice were placed in a single-chambered plexiglass apparatus (50 x 30 x 30) lined with aspen bedding, a nestlet, and food and water ad libitum for 24 hrs, a length of time demonstrated to be sufficient for the formation of residency behavior, and in which the arena becomes the individual’s territory [36–38]. Following this 24-hr period, residents were introduced to a same-sex intruder for an 8-min aggressive encounter period similar to previous studies [36, 39–41]. Intruders were only used for a single encounter, and otherwise had no previous aggression testing experience with unfamiliar individuals. During the encounter, defensive-aggressive barks were recorded using a single Emkay/Knowles FG series microphone (detection range: 10–120 kHz), with a 250 kHz sampling rate and 16-bit resolution, placed 30cm above the bottom of the chamber. Spectrograms were produced using a 512 fast Fourier transform in Avisoft SASlab pro (Avisoft Bioacoustics, Berlin, Germany) in order to identify barks, which appear as short, high-amplitude calls with an upside down U shape that begins and ends in the audible range for humans (Fig 2A) [25, 34]. Playback tracks were created using these spectrograms by manually extracting male and female bark calls that were confirmed by multiple observers, while removing all other USV call types. Calls could not be distinguished between the resident and the intruder during the encounter, thus both resident and intruder barks were used to construct playback tracks. Playback tracks included both male and female bark calls. Male and female bark calls are similar in structure and appear to have no contextual differences [25, 42] Playback tracks were 2 mins in duration and contained 120 ± 5 bark calls and followed the natural time pattern of what would be expected from an encounter, about one call per second. The output gain/volume was maintained across playback tracks. The ambient noise track control was 2 mins in duration and composed of a recording of the testing room with all lights off and no mice present using the same microphone described above. The ambient noise tracks were volume matched to the bark tracks during playback. We used eight unique tracks and assigned tracks to individuals randomly with each track used between 15–17 times, ensuring that no individual heard the same track more than once over the course of the two tests (to avoid habituation and maintain consistency). Playback amplitude was matched to the original bark amplitude produced by placing an Emkay/Knowles FG series microphone 30 cm away from the playback speaker and adjusting the output gain/volume to match the original recording [35].

### Pre-pairing playback test

Mice were first tested for response to bark playbacks as nonbonded, sexually naïve individuals. Mice were placed in the testing apparatus for 5–10 mins to habituate and enter all three chambers. Testing started with the mouse in the center chamber. 2-min playback preference tests were used with speakers at opposite ends of the apparatus behind wire mesh with one speaker playing a bark track and the other an ambient noise track concurrently. The side on which ambient noise versus the bark tracks were randomized to prevent habituation or bias due to side preference. Video and audio recordings were made of their behavior. We recorded time spent in the chamber closest to the bark speaker (“bark chamber”) as an approach score.

### Behavioral type and creation of ‘matched’ and ‘mismatched’ pairs

**Fig 1:**
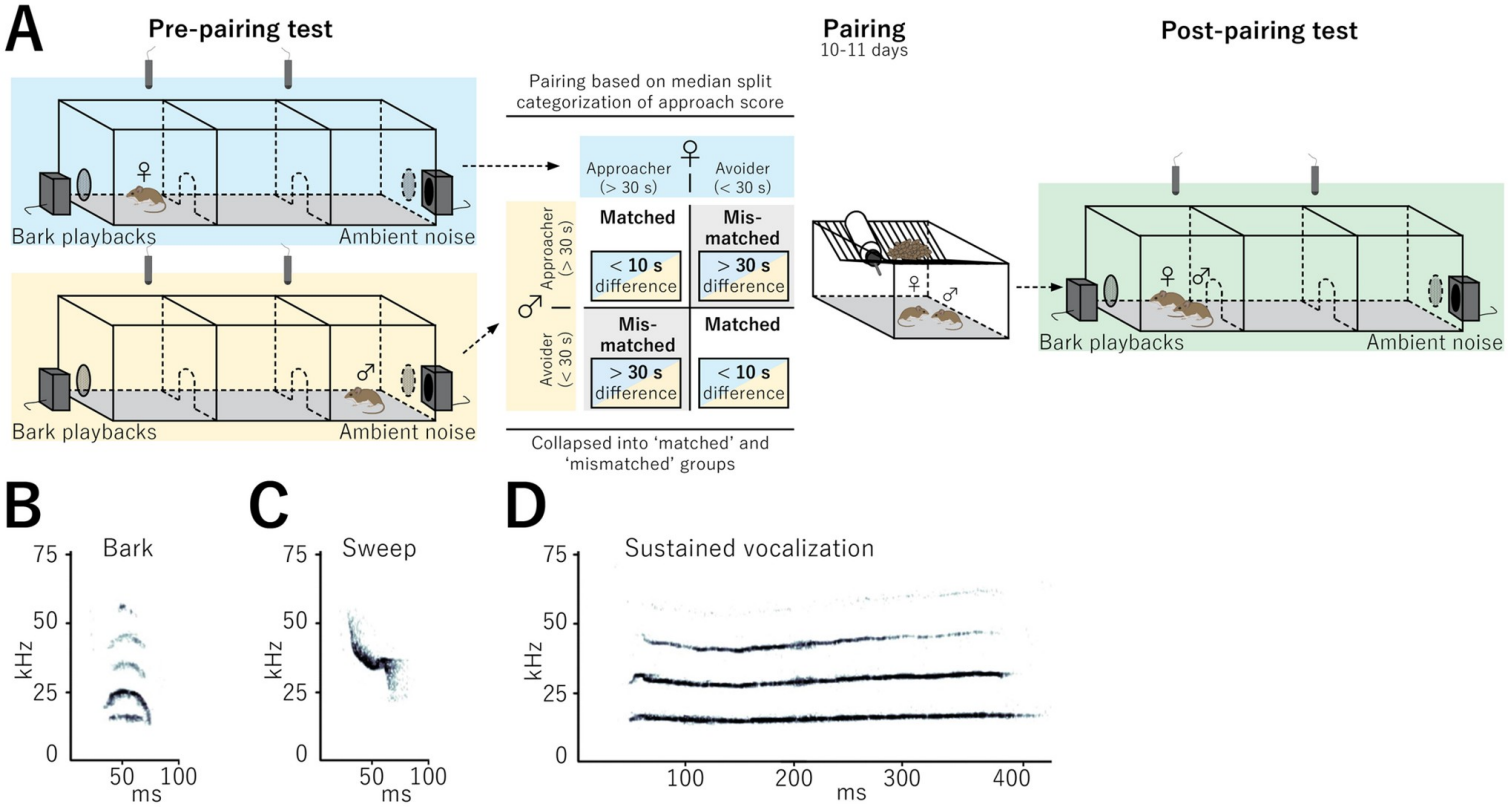
Overview of experimental design. **A**. Male and female mice were first tested individually for their response to bark playbacks. Based on their response they were paired using a 2 x 2 factorial design using level of approach and sex as factors. After 10–11 days post pairing, the pairs were tested again for their response as a dyad. Spectrograms showing the acoustic characteristics of **B)** Barks, **C)** Sweeps and **D)** Sustained vocalizations.

Following individual testing, mice were categorized as approachers or avoiders from a distribution of all individual responses to bark playbacks based on time spent in the bark chamber (Fig 1). Categories were defined using a median split (median = 30 s), where all male and female individual approach scores were pooled prior to calculation with mice above the median deemed approachers and mice below the median deemed avoiders. Mice (n = 62) were selectively paired following the pre-pairing test and allowed to cohabitate for 10–11 days prior to paired testing into one of four groups: 1) male approacher with female avoider (n = 7), 2) female approacher with male avoider (n = 11), 3) male approacher with female approacher (n = 5) and 4) male avoider with female avoider (n = 8). These groups were then collapsed into ‘mismatched’ (groups 1 and 2) and matched (groups 3 and 4) pair subtypes for analysis (see Results).

### Post-pairing playback tests

All mice underwent a second playback test to determine if pairing alters responses to bark calls. Pairs were retested 10–11 days after pairing (13–18 days after pre-pairing test). This time frame was used because at 7 days post-pairing, pairs display hallmarks of pair-bonding, specifically side by side contact and grooming and increased affiliative and decreased aggressive calling [35, 43] indicating that 10–11 days is sufficient for pair-bond formation. The playback procedure was the same as in the pre-pairing test except that paired mice were tested together as a pair. Both mice were placed into the central chamber and required to enter all three chambers prior to testing. Time spent in each chamber was scored for each mouse. Pairs were also scored for time spent together (both mice in the same chamber) and separate (different chambers).

### USV recording and analysis

Microphones were placed 30 cm from the apparatus floor with one microphone placed in the bark chamber and one microphone placed in the ambient noise chamber. Microphone channels were calibrated to equal gain (-60 dB noise floor) and WAV files were produced using RECORDER software (Avisoft Bioacoustics, Berlin, Germany). Recordings were made using a 250 kHz sampling rate with 16-bit resolution and spectrograms were produced with a 512 fast Fourier transform made using Avisoft SASLab Pro (Avisoft bioacoustics). SVs are low-bandwidth calls with low modulation, a peak frequency of 20 kHz and a duration of 100 to 500 ms for each individual syllable. Sweep calls are relatively short upward or downward frequency-modulated calls that can be either simple, lacking inflection, or complex, including inflections [25]. Both the total number of USV calls produced and the proportion of each USV individual call type produced relative to all call type production were analyzed within this dataset.

### Statistics

All statistics were analyzed using SPSS v 22 (IBM Corp, Armonk, NY, USA). We analyzed changes between the pre-pairing and post-pairing tests using a mixed ANOVA with group, sex, and pre-pairing similarity of pairs as factors. We analyzed USV call production by pairs using pair group (mismatched or matched) and similarity of pairs as factors. Tukey post hoc tests were used to determine differences between groups. We used correlations to test if behavior predicted USV call production and call type proportion. Pairs were used as a covariate in all appropriate analyses. All P-values were Holm-Bonferroni corrected for multiple comparisons where appropriate, including all correlations and post hoc tests.

## Results

### Individual response to bark playbacks and pair formation

**Fig 2:**
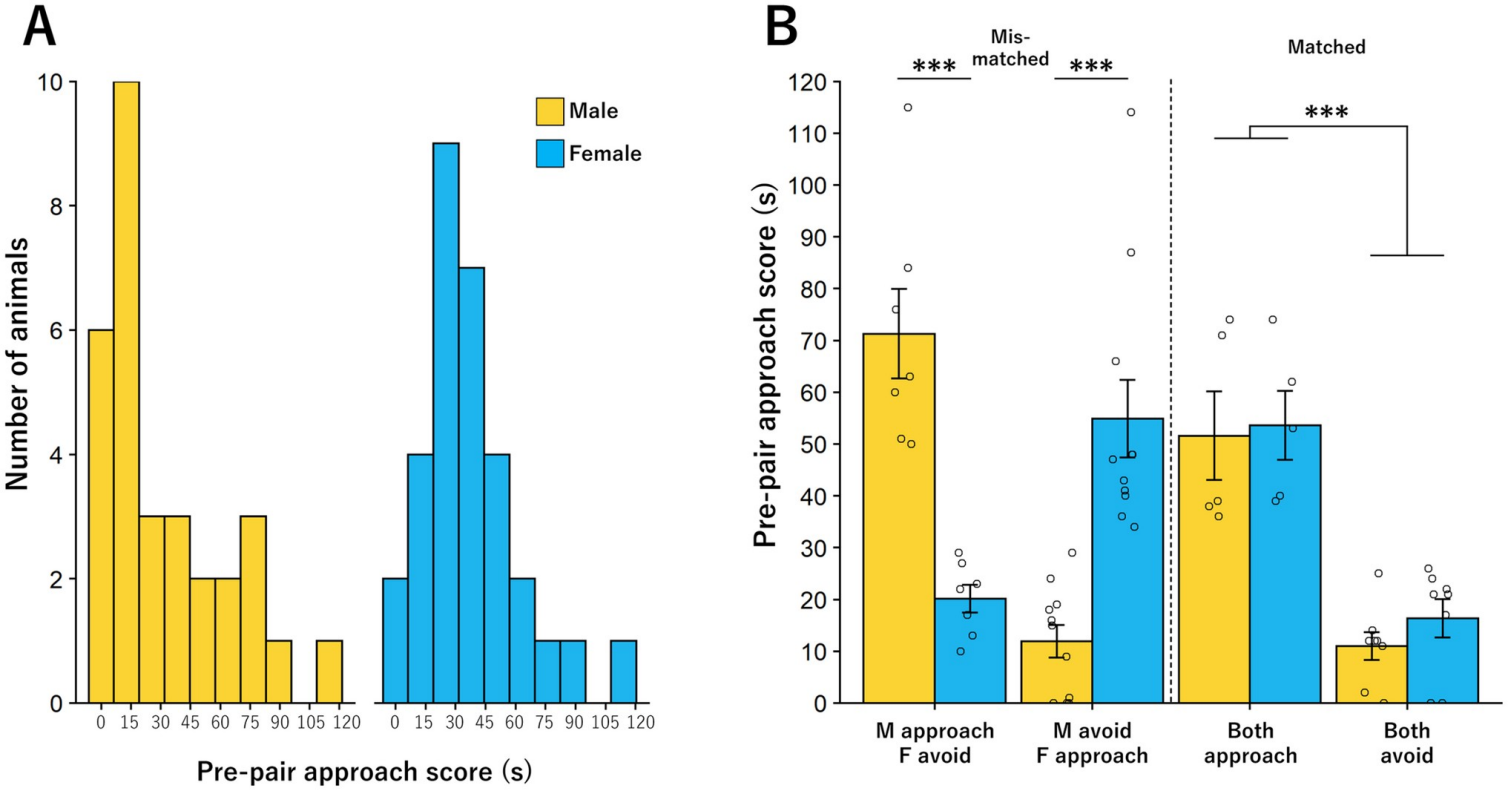
Individual responses to bark call playback. **A**. Histogram of pre-pair approach behaviors for males (yellow) and females (blue). **B**. Mean approach behaviors for each of the four paired. Significant differences were seen in approach behavior of mismatched but not matched pairs (* = p < 0.05). Bar graphs represent Mean +/- SEM.

During the pre-pairing test, mice showed a wide range of responses to bark playbacks. The range of time in the bark chamber was 0–115 out of 120 s (Fig 2A). Overall, as a group, mice did not show a preference for either the bark or the ambient noise chamber (ambient noise chamber: 36.42 ± 2.926 s, bark chamber: 36.19 ± 3.21, t(71) = 0.041, p = 0.967). Time spent in the bark chamber did not differ by sex, although there was a nonsignificant trend for females to spend more time in the bark chamber (males: 29.71 ± 4.28 s; females: 39.76 ± 3.87 s, ANOVA, F(1,80) = 3.032, p = 0.085). Similarly, the range of time spent in the ambient noise chamber ranged from 0–115 s and the average time in the ambient noise chamber did not differ by sex (males: 35.55 ± 3.994 s; females: 37.07 ± 4.196 s, ANOVA, F(1,72) = 0.066, p = 0.798; Fig 2A).

Based on initial response to the playback tracks individuals were paired in a 2 (high vs low) x 2 (male vs female) design to create matched and mismatched pairs of 4 types: male approacher/female avoider, male avoider/female approacher, both approach, both avoid. As expected based on assigned groups, there was a significant group by sex interaction in approach scores (ANOVA, F(3,54) = 24.013, p < 0.001, Fig 2B) with significant approach score differences between males and females in male approacher/female avoider, such that males had a higher approach score (male: 71.29 ± 8.67 s, female 20.14 ± 2.68 s, Students t-test, t(12) = 5.64, p < 0.001), whereas in male avoider/female approacher females had a higher approach score (male: 11.91 ± 3.17 s, female: 55.45 ± 7.41 s, t(20) = 5.40, p < 0.001). In contrast, there were no sex differences in both approach (male: 51.6 s ± 8.58, female: 57.40 ± 5.73 s, t(8) = 0.56, p = 0.59) or both avoid (male: 11.00 ± 2.70 s, female: 16.38 ± 3.68 s, t(14) = 1.17, p = 0.26). These groups were subsequently categorized by the difference in approach score within pairs: again, based on assigned groups, this difference was significantly higher in mismatched pairs than in matched pairs (male approacher/female avoider: 51.14 ± 8.02 s, male avoider/female approacher: 43.64 ± 7.98, both approach: 9.07 ± 4.06, both avoid: 5.88 ± 1.70: ANOVA, F(3,54) = 15.155, p < 0.001), reflecting our “approacher” and “avoider” classifications. Moreover, this analysis demonstrated that our four groups could be split into two homogenous subsets with male approacher/female avoider and male avoider/female approacher making up one subset of pairs (the “mismatched” subset) and both approach and both avoid making up the second homogenous subset (the “matched” subset). Thus, the groups were collapsed into these two overarching subsets for all subsequent analyses.

### Post-pairing response to playbacks

#### Approach and avoidance

**Fig 3:**
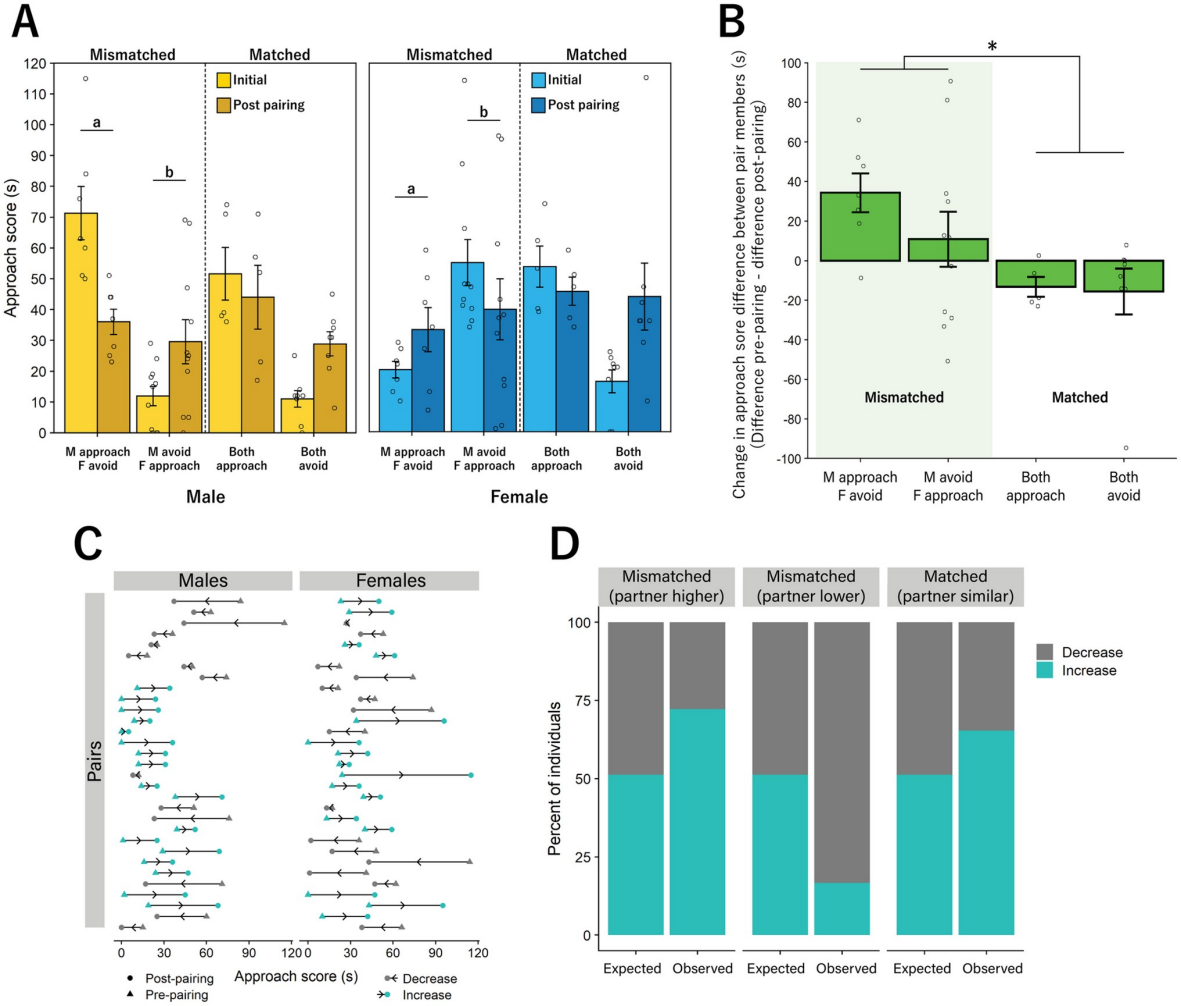
Pairs increased similarity in approach to bark call playback. **A**. Both male (left) and female (right) mismatched pairs altered their approach scores post pairing to become significantly more similar to their partner while matched pairs showed no significant difference in approach across the two time points. **B**. Mismatched pairs (left) showed a significant change in difference score between pairs from pre-pairing to post-pairing testing compared to matched pairs. **C**. Plots of individual changes in approach from pre-pairing test to post-pairing test. Increased approach scores are denoted by blue circles, while decreased approach scores are denoted by gray circles. **D**. The number of individuals that increased or decreased their approach score post pairing, compared to expected values based on random change in approach behavior. Members of mismatched pairs were more likely to systematically increase or decrease their approach score to match that of their partner, while matched pairs did not vary from expected outcomes. (a, b, * = p < 0.05). Significant lower-order effects were not indicated for any analyses. Bar graphs represent Mean +/- SEM.

Following pairing, a significant interaction was found between sex and group on approach score such that mismatched males and females altered approach behavior to be more similar to their partners. ‘Approachers’ decreased while ‘avoiders’ increased approach behavior, indicating that both behaviors can be altered (ANOVA, F(3,54) = 4.362, p = 0.008, Fig 3A). Using the overarching subsets of ‘mismatched’ and ‘matched’ we found a significant two-way interaction between group and pairing status on approach score, such that mice that were mismatched in their approach scores became more similar, and mice that were matched remained similar (F(1, 29) = 7.368, p = 0.011, Fig 3B). Whether individual changes in approach score were random or reflective of their initial approach score relative to their partner was tested via Chi-square. We found that individuals in mismatched pairs were more likely to increase or decrease their approach to match that of their partner than individuals who were in matched pairs (X^2^_2_ = 13.817, p < 0.0001, Fig 3D). As in the pre-pairing test, no preference was found for either the bark (26.71 ± 2.89 s) or ambient (39 ± 3.089s) noise chamber for individual mice during the second test (t(61) = 0.443, p = 0.66). There was also no difference in preference score based on pair group (F(3,54) = 0.281, p = 0.839).

#### Ultrasonic vocalizations

**Fig 4:**
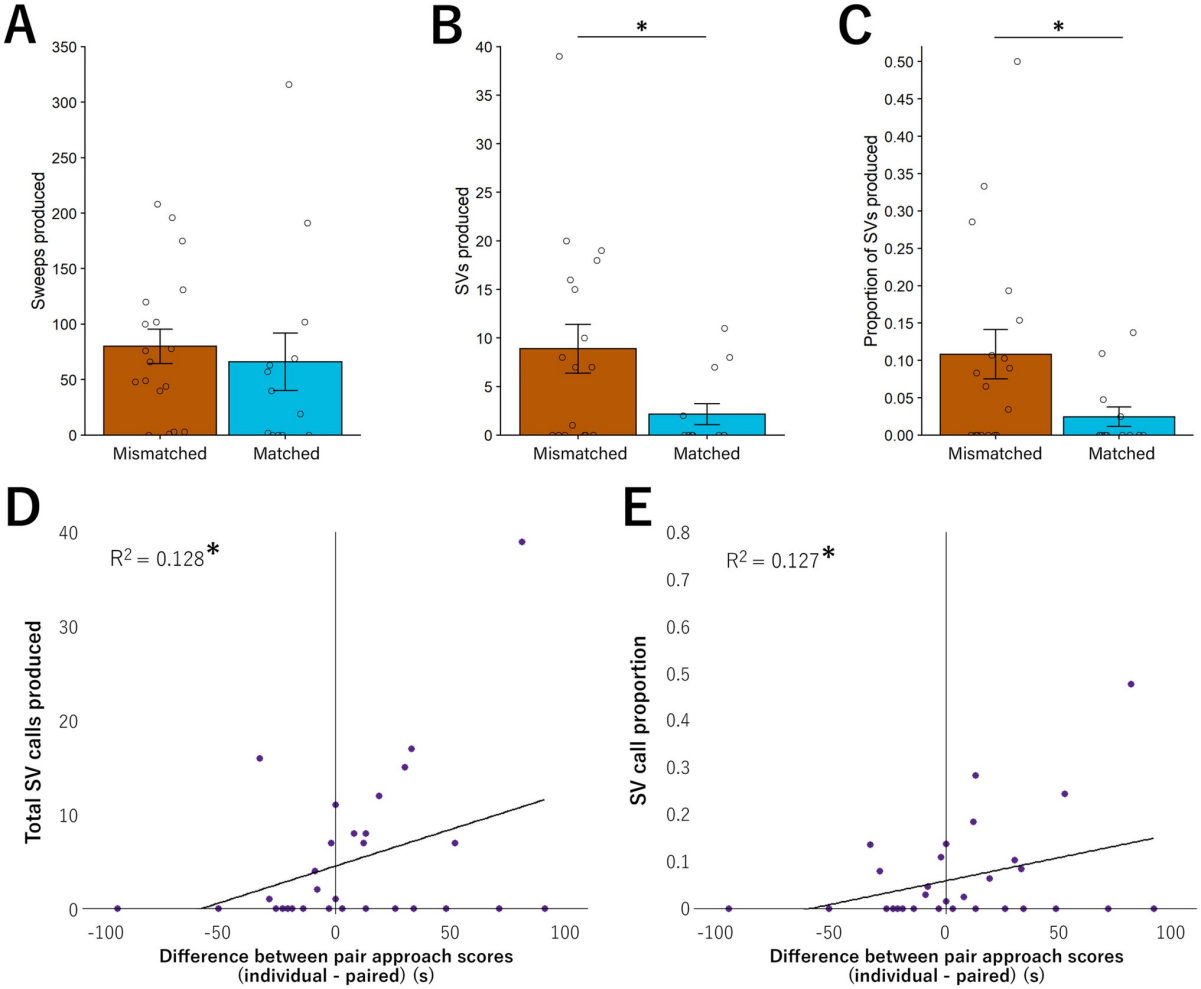
USVs correlated with behavioral coordination. **A**. Total number of sweeps were similar between mismatched and matched pairs **B**. Total number of SV calls increased in mismatched pairs compared to all other groups. **C**. The proportion of SV calls as a function of total calls was greater in mismatched pairs compared to matched pairs. **D**. Pairs with greater similarity in post-pairing approach scores produced more SVs as a dyad. Pairs that became more similar (pre-pairing approach score difference—post-pairing approach score difference) as denoted by positive numbers on the x-axis, produced more total SV calls as well as **E**. a significantly greater proportion of SV calls to total USV calls produced (* = p < 0.05). Bar graphs represent Mean +/- SEM.

During the post-pairing test, total USV call production did not differ between mismatched (87.0 ± 16.37 USVs) and matched (66.08 ± 26.30 USVs) pairs during bark playbacks (ANOVA, F(1,29) = 0.404, p < 0.53). Specifically, sweep production was similar between mismatched (80 ± 15.73) and matched (66.08 ± 25.81) pairs (ANOVA, F(1, 29) = 0.239, p = 0.629, Fig 4A). However, the total production of SV calls was greater in mismatched pairs (7 ± 2.38) compared to matched pairs (2.23 ± 1.06; ANOVA F(1, 29) = 4.966, p = 0.0338, Fig 4B). As relative proportions of USV types have been associated with pair affiliation [44], including proportion of SVs to total USVs [32], we also calculated a ratio of pair SVs to total pair USVs. Mismatched pairs (9.25 ± 0.28%) produced a greater proportion of SVs as a function of total USVs compared to matched pairs (2.57 ± 1.27%; ANOVA, F(1, 29) = 6.03, p = 0.02, Fig 4C).

Total USVs and proportion of SVs correlated with several behavioral measures. Importantly, increased similarity in approach score by pairs correlated with more total SVs produced as a dyad (t(29) = 2.071, Pearson’s r = 0.359, p = 0.047 Fig 4D) and a greater proportion of SVs compared to all USVs (t(29) = 2.056, Pearson’s r = 0.357, p = 0.049, Fig 4E).

## Discussion

Behavioral responses can be altered by changing an individual’s social environment [45], such as when a pair-bond develops. Here we describe for the first time in the monogamous California mouse evidence of approach behavior towards an aversive stimulus that is altered in an emergent, context-dependent fashion by social-bonding leading to a possible ‘pair personality’ [46], whereby pairs with mismatched behavioral types become more similar following pairing, while matched pairs maintain their similarity. We further show that behavioral convergence is correlated with increased SVs as a proportion of total USVs, indicating a potential role of USVs in behavioral coordination. With this study, we provide a novel monogamous mammalian model of behavioral plasticity due to pair-bonding that provides insights into how and why individuals become more similar in their behavior due to social change. This model will allow for future studies to determine the longevity of these changes, and the neural mechanisms that underlie this behavioral convergence.

### Behavioral convergence following pair-bonding

California mouse pairs display variation in their approach during a territorial defense paradigm [19]. However, how pair bonding alters these behavioral responses remains unknown. This is a question of great interest, because to maximize survivability and reproduction within a pair bond, pairs need to coordinate their behavior [5]; whether pair bonding mammals, and California mice in particular, change their individual responses to stimuli in the presence of a partner that has a different response to a challenging stimulus or whether individuals simply maintain their responses (and potentially mate with others who have complimentary responses) has not been tested. To test this, we focused on approach response to aggressive bark playbacks of intruding conspecifics during the time when bonding was initiated but prior to pup birth. We found that mismatched pairs changed their approach to bark playbacks to become more similar after pair-bonding, while matched pairs remained similar, regardless of the individuals’ initial type. Because we measured behavioral responses prior to and following pair-bond formation, we provide evidence that similarity can occur independently of assortative mating. The behavioral convergence may relate to the ongoing formation of a pair bond, however, pair bonds have typically been formed by the time that the playback challenge was conducted [32, 35].

We did not test individuals with a non-bonded partner such as a cage mate, however, because in the wild California mice would not inhabit a territory with a partner with whom they did not share a pair-bond and defend a territory and thus this would lack ecological relevance [47, 48]. Our findings are consistent with evidence that newly paired convict cichlids make post-pairing adjustments to become more similar along a proactive-reactive axis encompassing boldness, that is associated with increased fitness [49]. However, extending these findings to include species that form social bonds beyond pair bonding would be an exciting future direction.

This finding differs slightly from previous results in our lab. Notably, in response to a live intruder in the lab, established California mouse pairs show different behavioral responses, namely a divided or joint investigation of the pair [19]. However, in this study, we found a move towards synchronicity across the board, where partners tended to stick together in response to playbacks. This is likely because pairs in the current paradigm were not in a home territory, causing them to remain closer together throughout the test. This also makes sense because this was a neutral arena (no residency effect formed) with no nesting material or igloo to defend and because the pairs did not have pups (a significant driver towards pairs acting separately in previous tests) there is no reason for one individual to remain behind and protect resources in response to a potential challenge [50]. This could also be due to the use of bark calls as the stimulus. Bark calls generally occur during aggression [25] and may signal slightly different information than SVs, such as an ongoing conflict or alarm call in the area that needs to be investigated. This is an intriguing future direction as it begins to tease apart the function of calls and how they are used to drive decision making by pairs. As such, this study suggests that pairs show behavioral convergence, particularly in a neutral arena, but leaves open the idea that context (home or away), as well as degree of pair bonding are important drivers of pair behavior.

An emerging question regarding monogamous, biparental species is how they are capable of both maintaining bonds and coordinating labor after offspring are born. Pair-bonded California mice face many challenges including foraging, pup care, mate attendance, and territorial defense, and coordination may promote task efficiency and/or pair-bonding [51, 52]. Increased similarity may afford greater cooperation within the pair-bond to complete tasks related to territorial defense. This would align with data in voles [5] and California mice [19] showing that bonded males and females can participate in the same tasks. Alternatively, coordination may strengthen the pair-bond as indicated by increased contact time including huddling and grooming [48]. Long term maintenance of the pair-bond may explain pair coordination since pair duration can be associated with reproductive success [53], and while California mouse pairs display hallmarks of pair-bonding including side by side contact, reduced aggression, increased affiliation and increased affiliative USV calls by 7 days following pairing [35, 43], we expect that pair-bond maintenance is an ongoing process. We speculate that the optimal coordination strategy depends on the intensity and type of challenge. Future research will determine if increased similarity is due to increased pair coordination by exposing pairs to a challenge in which a different coordinated strategy becomes advantageous.

### Ultrasonic vocalizations

Are pairs communicating with each other and could this influence development of potential emergent “pair personalities”? In this experiment, pairs produced more USVs than individuals, suggesting that two possible reasons that USVs are expressed are 1) an internal state change occurs when near their partner that does not alter their partner’s behavior, or 2) an attempt to influence changes in the partner’s behavior to achieve an efficient unit for raising young and defending a territory. While other explanations for these calls may exist, we have also previously seen that increased pair calling in a first encounter drives behavioral convergence in subsequent encounters of the same kind [19]). These data taken together, particularly that mismatched pairs produce the most calls and that this correlates with behavioral convergence, would suggest that USV production by pairs helps to coordinate pair behavior, and helps drive those behaviors towards synchronicity. Additionally, since the proportion of SVs to total USVs correlated with increasing similarity in approach behaviors between pairs, USVs may mediate behavioral coordination within an aggressive context. Importantly, these calls were not produced as animals were huddling together and no huddling behavior occurred during this paradigm indicating that these calls likely are not due to simple affiliation but instead are helping to drive coordination. Across species, communication is important for coordinating social behaviors, such as responses to threats [54], territorial defense [55–57], and information sharing [58–61]. As such, communication may play an important role in coordinating behavioral similarities within pairs.

### Conclusion

In collective behaviors, individuals often follow simple rules resulting in group-level characteristics difficult to predict based on individual-level behaviors. We provide evidence in monogamous rodents that formation of a pair bond can lead to conformity in social behavior. Overall, we found that nonparental, pair-bonded California mice showed changes in responses to aversive stimuli leading to greater similarity in behavior that we speculate could be adaptive when jointly defending a territory. Furthermore, this increased behavioral similarity corresponded with increased communication in the form of SVs, indicating, for the first time in a monogamous rodent, the potential importance of vocal communication for coordinating behavior between mates in order to increase behavioral similarity. Our research adds to a growing body of literature underscoring the importance of accounting for individual-level variation and its role in producing emergent variation at the level of the pair.
